# Unilateral Renal Agenesis: Prenatal Diagnosis and Postnatal Issues

**DOI:** 10.3390/diagnostics15131572

**Published:** 2025-06-20

**Authors:** Waldo Sepulveda, Amy E. Wong, Gabriele Tonni, Gianpaolo Grisolia, Angela C. Ranzini

**Affiliations:** 1 Fetal Imaging Unit, FETALMED—Maternal-Fetal Diagnostic Center, Santiago 7591047, Chile; 2 Department of Maternal-Fetal Medicine, Palo Alto Foundation Medical Group, Mountain View, CA 94301, USA; amy.wong@gmail.com; 3 Department of Obstetrics and Gynecology, Istituto di Ricovero e Cura a Carattere Scientifico (IRCCS), AUSL Reggio Emilia, 42122 Reggio Emilia, Italy; gabriele.tonni@ausl.re.it; 4 Department of Obstetrics and Gynecology, Carlo Poma Hospital, ASST Mantova, 46100 Mantua, Italy; grisoliagp@gmail.com; 5 Division of Maternal-Fetal Medicine, Department of Obstetrics and Gynecology, The MetroHealth System/Case Western Reserve University, Cleveland, OH 44109, USA; acranzini@gmail.com

**Keywords:** empty renal fossa, fetal ultrasound, kidney, prenatal diagnosis, renal agenesis, solitary kidney, unilateral renal agenesis

## Abstract

Unilateral renal agenesis (URA) is a urinary tract congenital anomaly characterized by a congenital absence or early developmental arrest of only one kidney. In the presence of a normal contralateral kidney, URA is typically considered a condition of minimal clinical significance as the solitary kidney often undergoes hypertrophy and can sufficiently perform the needed renal function after birth. However, postnatal studies suggest that URA has a significant association with other urinary and extra-urinary anomalies and may have implications for long-term health. This descriptive review focuses on the perinatal aspects of URA, emphasizing the main ultrasound findings to establish the prenatal diagnosis and to guide perinatal management. The pediatric implications of this diagnosis, particularly the high prevalence of long-term complications including hypertension, proteinuria, and a decreased glomerular filtration rate, are also briefly reviewed. URA is consistently associated with other ipsilateral urogenital anomalies. In females, there is a significant association with uterine anomalies that has significant implications for subsequent reproductive function. In males, the prevalence of both urinary and genital anomalies is also increased, which may also have implications for future fertility. Prenatal ultrasound offers the possibility of early diagnosis and parental counseling, which may result in timely intervention to reduce contralateral renal damage, prevent severe urogenital manifestations and co-morbidities, and improve fertility and the quality of life.

## 1. Introduction

Urinary tract anomalies are one of the most frequent findings on routine second-trimester ultrasound screening for structural malformations [1,2,3]. Several types of these anomalies have a dismal prognosis leading to perinatal death, especially those associated with severe oligohydramnios and pulmonary hypoplasia, as in bilateral renal agenesis (BRA) and lower urinary tract obstruction, or when severe bilateral anomalies causing a serious impairment of renal function are present [4,5,6]. However, the vast majority of other urinary tract anomalies are associated with a favorable prognosis, especially when a normal contralateral kidney is present. Among them, isolated unilateral renal agenesis (URA), despite its variability in prognosis, is often considered a benign condition since the contralateral kidney can compensate for this and typically performs the needed renal function. Indeed, this condition can be entirely asymptomatic and in many cases can remain undiagnosed throughout life [7].

These considerations have led to the impression that URA is not necessarily a clinically important condition [8,9,10]. However, clinical evidence from pediatric long-term outcome studies has shown that there are several issues that are often overlooked during prenatal and neonatal counseling, including the association with urinary and non-urinary anomalies that may affect individuals with URA during childhood or later [11,12]. These issues should also be discussed with prospective parents, as they might have additional implications for subsequent medical evaluations and pediatric health care.

This descriptive review aims to discuss the main ultrasound diagnostic features, differential diagnosis, and subsequent management of URA when detected prenatally. Representative images of the main antenatal findings are presented, and the clinical implications during childhood, puberty, and adulthood are briefly discussed.

## 2. Prenatal Diagnosis

According to Edith L. Potter [13], the pioneer of perinatal pathology, URA occurs in 2.6–20/10,000 individuals, is more prevalent in males, and more often affects the left kidney. A more recent review based on ultrasound diagnosis confirmed a similar incidence of approximately 1/2000 [11]. Currently, the prenatal diagnosis of URA is primarily based on obstetric ultrasound through the identification of an absent kidney in the corresponding renal fossa [14,15]. The diagnosis is, however, frequently missed because it can be difficult to recognize that a kidney is absent in the presence of a normal amniotic fluid volume, normal bladder, and normal contralateral kidney [14,15]. In this setting, it is often presumed that both kidneys are present and anatomically normal without a thorough evaluation of both renal fossae. Indeed, URA is a condition that is diagnosed only when a focused ultrasound examination of both renal fossae is performed.

### 2.1. Prenatal Ultrasound of Normal Kidneys

**Figure 1:**
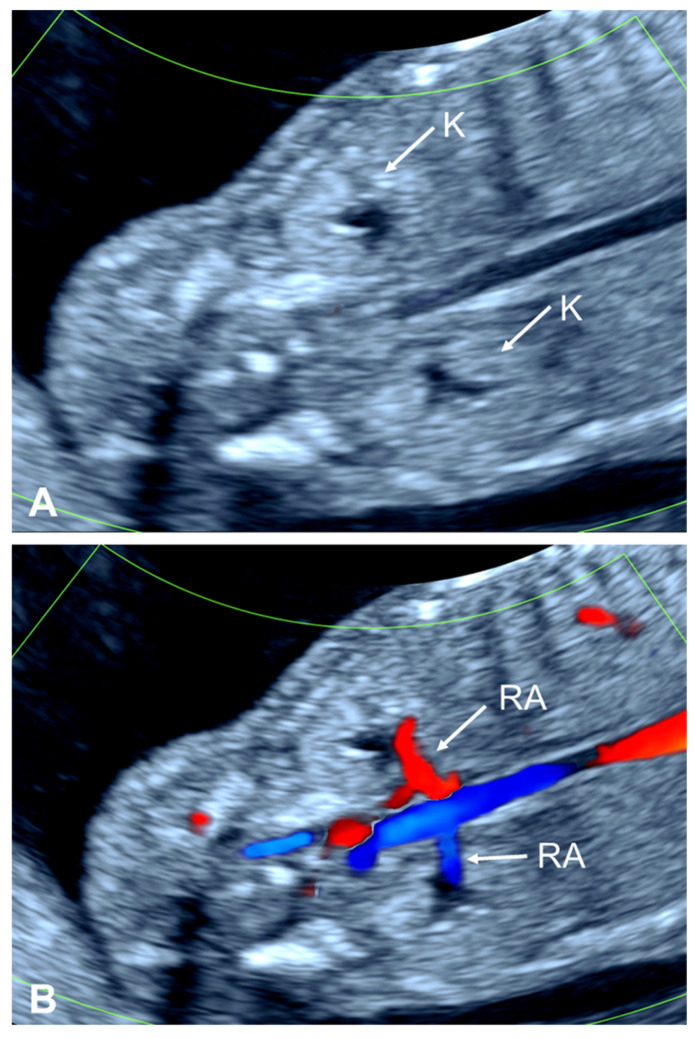
The transvaginal posterior coronal view of the kidneys at 13 weeks’ gestation. (**A**) The kidneys are visualized on either side of the spine and have pelvises that are centrally placed in the renal parenchyma. (**B**) The renal arteries are clearly depicted with superimposed color flow imaging. The descending aorta and upper portion of the aortic bifurcation are also shown. K, kidney; RA, renal artery.

The fetal kidneys are normally located retroperitoneally at the level of the lower thoracic and upper lumbar spine, usually between T12 and L3 [16]. Prenatally, they appear as isoechoic, ellipsoid, bean-like structures in the lumbar paravertebral area resting on the psoas muscles. In normal conditions, both kidneys can be visualized with transvaginal ultrasound from the late first trimester onwards (Figure 1). The visualization of the kidneys in the first trimester is easier in a coronal view with the spine in the center of the image. Throughout pregnancy both fetal kidneys have similar echotextures and positions, although the right kidney may be located slightly caudal to the left kidney due to the presence of the liver predominantly in the right hemiabdomen. The cranio-caudal (longitudinal) diameter of the kidney is larger than the transverse and antero-posterior diameters, with no differences in size between the right and left kidneys. Reference ranges for all three diameters and renal volume are available from several authors [17,18,19,20].

**Figure 2:**
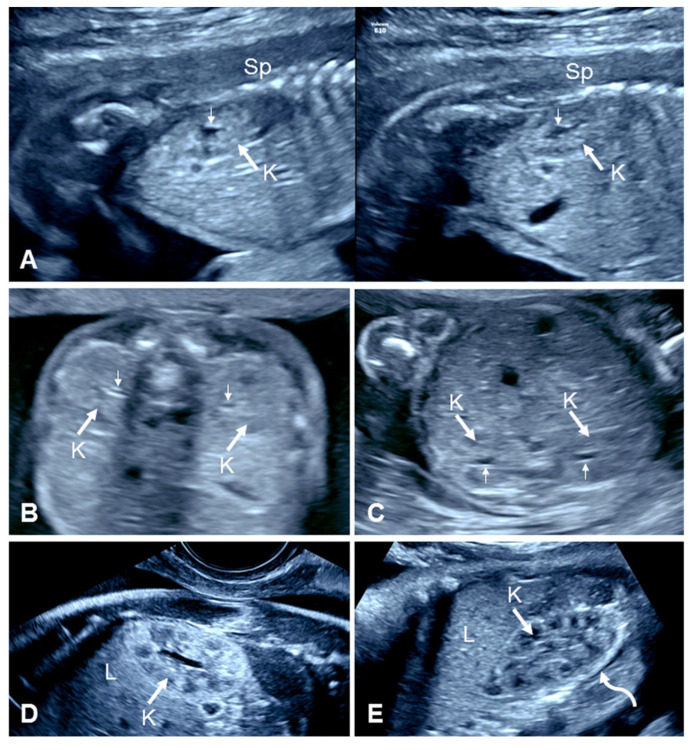
Second- and third-trimester ultrasound views of normal fetal kidneys. (**A**) Split-screen transabdominal ultrasound at 21 weeks’ gestation shows normal right (**left panel**) and left (**right panel**) kidneys in their normal position. Note the renal pelvises surrounded by normal renal parenchyma (small arrows). (**B**,**C**) Transabdominal axial views of the fetal abdomen show the fetal kidneys in second-trimester fetuses in the prone and supine positions, respectively. Note acoustic shadowing from the spine when the fetus is in the prone position. Small arrows denote the renal pelvises. (**D**) Transvaginal ultrasound at 23 weeks’ gestation shows normal kidney, renal pelvis, and pyramids. (**E**) The transabdominal ultrasound of the fetal kidney at 36 weeks’ gestation shows normal renal parenchyma and prominent echogenic perirenal fat (curved arrow), which makes the kidney easily identifiable in the third trimester. K, kidney; Sp, spine; L, liver.

During the second and third trimesters, an ultrasound examination of the fetal abdomen in axial views at the level of the corresponding renal fossae can easily display the kidneys, which have a characteristic central anechoic area representing the renal pelvis that is surrounded by renal tissue (Figure 2) [3,21]. Nevertheless, it is easier to evaluate the kidneys in sagittal planes with the spine in the 12 o’clock position. Care should be taken to ensure that the renal pelvises are imaged. Coronal images are helpful for identifying the renal arteries with color flow imaging and for ensuring that the collecting system of each kidney is centrally located [22]. Within the renal parenchyma, several anechoic and hypoechoic areas representing the calyces and renal pyramids, respectively, are progressively visualized towards the third trimester. In late pregnancy, the outline of the kidneys is enhanced by the presence of perirenal fat [21]. This is not the case in second-trimester fetuses, in which the isoechoic renal tissue may even resemble the fetal large bowel, making the kidneys more difficult to identify during the routine mid-trimester scan than in the third trimester. Nevertheless, focused axial views of the kidneys should be routinely obtained as part of the fetal second-trimester anatomy scan for detecting urinary tract dilation [22]. Sagittal and/or coronal views can also provide adequate views of the kidneys and may be superior when the fetus is supine or in a lateral position when the lumbar vertebrae can obscure the more distally located kidney or when the kidneys/renal pelvises are otherwise difficult to image. In addition, several technical factors can also impair the visualization of the kidneys, such as maternal obesity, previous maternal abdominal surgery, large myomas, or oligohydramnios.

### 2.2. Prenatal Ultrasound in Unilateral Renal Agenesis

The ultrasound findings that suggest the diagnosis of URA in the presence of a normal contralateral kidney combines the recognition of direct signs, such as the visualization of an empty renal fossa in the absence of an ectopic or horseshoe kidney, and indirect signs such as the identification of an ipsilateral ‘lying down’ adrenal gland, contralateral compensatory renal hypertrophy, and the non-visualization of the ipsilateral renal artery on color flow imaging [9,10,14,15]. Whenever an empty renal fossa is detected, the fetal lower abdomen and pelvis should be carefully evaluated to exclude a pelvic kidney. These can be difficult to find due to shadowing from the fetal iliac wings and spine. In fetuses with URA and a normal contralateral kidney, both the fetal bladder and amniotic fluid volume appear normal. The prenatal assessment of renal function with a biochemical analysis of amniotic fluid, fetal urine, and fetal serum markers is therefore of no utility.

#### 2.2.1. Empty Renal Fossa

**Figure 3:**
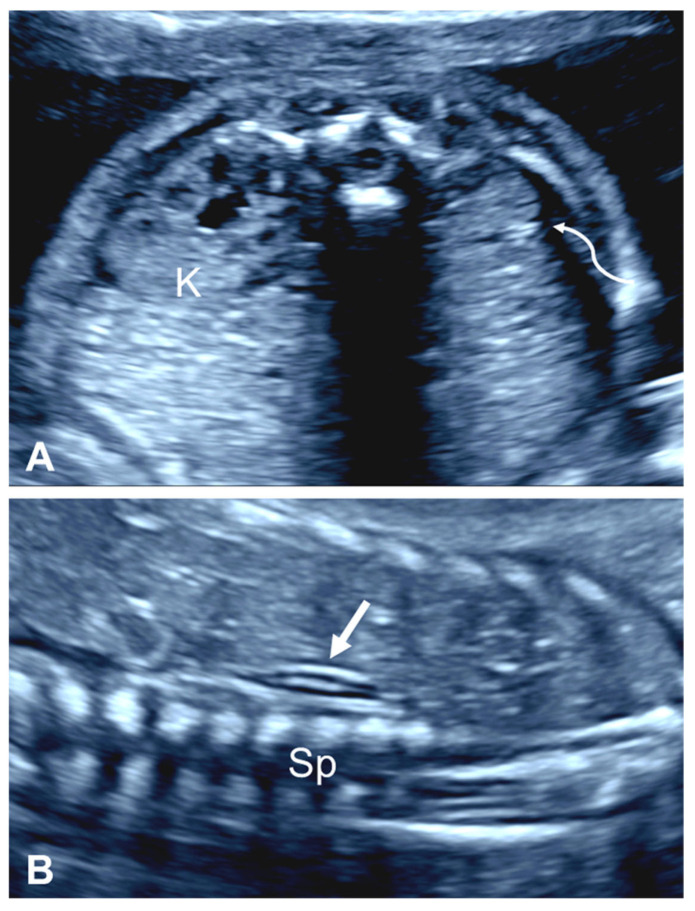
An empty renal fossa in a fetus with unilateral renal agenesis at 25 weeks’ gestation. (**A**) The axial view of the fetal abdomen shows a normal kidney (K) located in the renal fossa and the contralateral empty renal fossa (curved arrow). Note that while there is some isoechogenic tissue in the empty renal fossa, a renal pelvis is not identified. (**B**) The sagittal view of the empty renal fossa shows the adrenal gland lying over the spine. A flattened ‘lying down’ adrenal gland is clearly depicted (arrow). Sp, spine.

**Figure 4:**
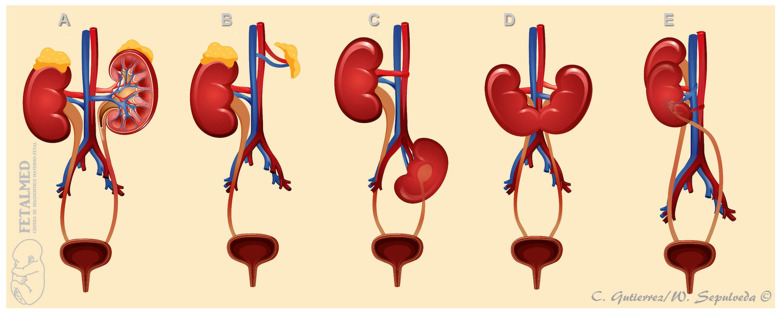
A schematic representation of different conditions presenting with an empty renal fossa. (**A**) Normal. (**B**) Unilateral renal agenesis. The ‘empty’ renal fossa is indeed occupied by a ‘lying down’ adrenal gland. (**C**) Ectopic pelvic kidney. (**D**) Horseshoe kidney. (**E**) Crossed fused ectopic kidney. The adrenal glands are not shown in (**C**–**E**) for illustrative purposes only.

The most important, although nonspecific, prenatal ultrasound feature of URA is the absence of one kidney in the corresponding renal fossa, a condition commonly referred to as an ‘empty renal fossa’ (Figure 3). However, there are several other renal disorders that can present with an empty renal fossa including ectopic kidney (most commonly a pelvic kidney), renal ptosis or ‘floating kidney’, horseshoe kidney, and crossed fused renal ectopia (Figure 4) [14].

The pathophysiologic events accounting for an absent kidney include primary agenesis [7], renal aplasia [23], and the early developmental arrest of a unilateral multicystic dysplastic kidney (MCDK) [24]. Care should be taken to distinguish the loops of the large bowel and the adrenal glands from renal tissue [21] and, more importantly, to exclude the presence of an ectopic kidney, which is typically located in the fetal pelvis [25].

By far, the two main causes of an empty renal fossa are URA and pelvic kidney; less commonly, a horseshoe kidney, crossed fused renal ectopia, or a non-pelvic ectopic kidney may occur [26]. In 1990, Sherer et al. [27] were the first to report the prenatal diagnosis of URA in two third-trimester fetuses presenting with a unilateral empty renal fossa and prominent but structurally normal contralateral kidney. In the same year, Jeanty et al. [28] reviewed the prenatal ultrasound findings in 6 fetuses with a unilateral empty renal fossa, all detected after 28 weeks’ gestation. Table 1 summarizes the studies examining the diagnosis of fetal empty renal fossa in the second and third trimesters of pregnancy [28,29,30,31,32]. Of a total of 173 cases, 74 (43%) had URA, and 70 (40%) had a pelvic kidney.

#### 2.2.2. ‘Lying Down’ Adrenal Gland

In normal conditions, the adrenal (suprarenal) gland is located horizontally overlaying the upper pole of the kidney. On ultrasound, this organ is identified as an elongated, ovoid structure that contains an echoic medulla and a long hypoechoic cortex [33]. In the fetus, it is visualized in the axial section above the kidney as a flattened Y-shaped structure extending medially to laterally in the upper renal fossa [33]. It can also be visualized in coronal views as a hypoechoic triangular structure superior to the kidney.

When one or both kidneys are not present or ectopic in location, the ipsilateral adrenal gland takes a characteristic position running in the cranial–caudal direction, parasagittal to the thoraco-lumbar spine, a sign which has been termed the ‘lying down’ adrenal gland [34]. However, a normal shape and location of the adrenal gland have been documented in fetuses with URA [35]. There is also the possibility that the discoid shape of the fetal adrenal gland can mimic the presence of the kidney [21,36].

#### 2.2.3. Contralateral Renal Hypertrophy

The compensatory renal hypertrophy of one normal kidney can occur in three clinical settings: (i) when the contralateral kidney is congenitally absent, as in the case of URA; (ii) when the contralateral kidney has no renal function, as in the case of unilateral MCDK or noncystic renal dysplasia; or (iii) when one kidney has been removed surgically, as in the case of nephrectomy due to a renal tumor or organ donation [37]. Only the first two conditions occur in utero. Multiple studies have reported larger sonographically measured renal lengths of solitary kidneys in cases of URA and unilateral MCDK, particularly in the third trimester, suggesting that the rate of hypertrophy accelerates as pregnancy progresses [38,39,40,41]. Assessments of renal hypertrophy based on an anterior–posterior-to-transverse diameter ratio greater than 0.9 [31] and renal volumes [42] have similarly supported compensatory hypertrophy. In addition, fetal autopsy specimens have shown a higher kidney weight [43], and contralateral renal hypertrophy is a frequent finding in children with URA, as demonstrated using different imaging modalities including ultrasound, radioisotope venography, computerized tomography, and magnetic resonance imaging (MRI) [44].

**Figure 5:**
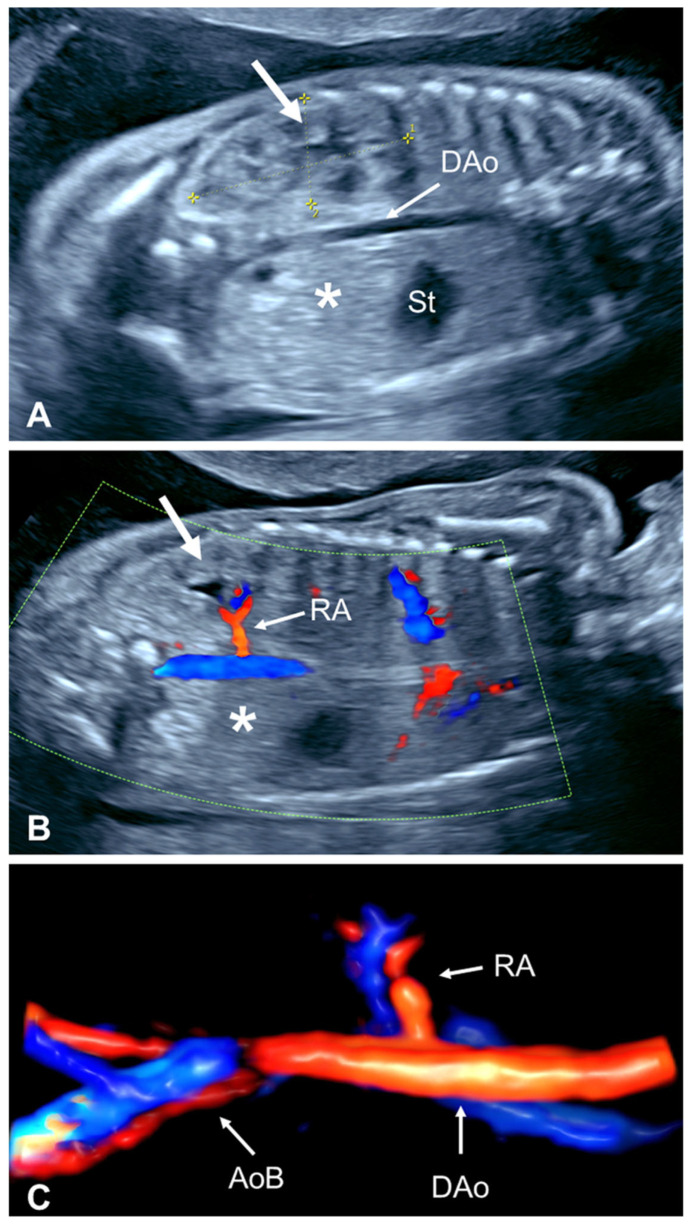
Prenatal ultrasound in unilateral renal agenesis. (**A**) The conventional two-dimensional transabdominal posterior coronal view at 22 weeks’ gestation shows a solitary kidney (arrow) measuring 36 × 17 mm (between calipers *1 and *2), above the 95th percentile according to the reference range [19]. The contralateral renal fossa is empty (asterisk). (**B**) Color flow imaging shows the solitary renal artery at the posterior coronal view. Note the contralateral empty fossa with an absent renal artery. The solitary kidney is denoted by an arrow. (**C**) Three-dimensional sonoangiography shows the presence of only one renal artery. The descending aorta and aortic bifurcation are clearly seen. DAo, descending aorta; St, stomach; RA, renal artery; AoB, aortic bifurcation.

Although several reference ranges for fetal renal biometry are available, subjective assessment with the impression of a larger-than-normal kidney may be the first clue leading to the diagnosis of URA after an empty renal fossa is found following an attempted evaluation of the contralateral kidney (Figure 5A) [27]. Conversely, if there is no compensatory hypertrophy of the normally located kidney in the third trimester, this suggests that there may be an additional kidney in an unusual location. Of note, the reason why a solitary kidney undergoes hypertrophy in utero in the presence of a normal placental clearance has not been yet elucidated [45].

#### 2.2.4. Absent Ipsilateral Renal Artery

The renal arteries branch at the level of the renal fossae directly from the abdominal aorta at a nearly 90-degree angle. Due to their size, the fetal renal arteries can be easily identified using color flow imaging on the posterior coronal plane of the middle abdomen. The renal arteries are located approximately halfway between the diaphragm and pelvic aortic bifurcation of the abdominal aorta [46]. Fetuses with BRA display the abdominal aorta and aortic bifurcation, but renal arteries are not identified [47,48]. Similarly, in fetuses with URA, only one renal artery is identified (Figure 5B,C) [14,15,21]. Theoretically, the arterial diameter of the remaining renal artery should be larger due to the compensatory increase in blood flow into the normal contralateral kidney. However, this feature has not been studied prenatally or postnatally. Color flow imaging could also be useful to detect a fetal pelvic kidney, as in these cases the ipsilateral renal artery is present but branches more caudally into the lower abdomen and pelvis to perfuse the ectopic kidney. Although the ipsilateral adrenal or the gonadal artery could potentially be mistaken as a renal artery in some fetuses with URA, the smaller diameter of these arteries makes it difficult to confound them with a renal artery.

## 3. Prenatal Evaluations

### 3.1. Prenatal Imaging Assessment

Several syndromes and genetic conditions have been described in association with BRA, including branchio-otorenal syndrome, Fraser syndrome, sirenomelia, and VATER syndrome (Vertebral anomalies, Anal atresia, Tracheo-Esophageal fistula, Renal anomalies)/VACTERL association (Vertebral anomalies, Anal atresia, Cardiac anomalies, Tracheo-Esophageal fistula, Radial and Renal anomalies, and Limb anomalies), among others [49,50,51]. Because BRA is a congenital anomaly incompatible with extrauterine life and, therefore, the termination of affected pregnancies is often performed, whether an associated syndrome is present often remains unknown.

Similarly, URA is also a prominent feature of several non-lethal genetic syndromes, some of which can be simultaneously diagnosed prenatally in fetuses presenting with multiple congenital anomalies [49,50]. The most common associated syndromes include VATER syndrome/VACTERL association [52] and occasionally CHARGE syndrome (Coloboma, Heart disease, Atresia choanae, growth Restriction, Genital anomalies, and Ear anomalies) [53,54]. These two conditions might present prenatally with several findings affecting multiple systems, some of which are indeed more evident than the congenital absence of one kidney itself. Due to the diversity of organ systems involved and variable phenotypic penetrance, the associations of these malformations are sometimes difficult to put together, and fetal MRI and/or specific molecular genetic testing may be helpful adjunct diagnostic techniques. However, some cases can only be definitively diagnosed following a thorough evaluation after delivery or postmortem studies [52,53]. Syndromic and non-syndromic conditions involving genital malformations associated with URA, usually undetected prenatally, are discussed below.

A different clinical scenario is the prenatal detection of URA as an isolated finding. In these cases, a clear distinction between isolated URA, which presents as a ‘solitary kidney’, and unilateral MCDK, which presents as a ‘solitary functional kidney’, should be made. In URA there is only one kidney due to a congenitally absent contralateral kidney, whereas in unilateral MCDK there are two kidneys, the affected one being fully non-functional and therefore easier to diagnose prenatally by ultrasound than URA. Many studies in the prenatal and postnatal literature have grouped these cases together, although prognostic differences between URA and unilateral MCDK have been recently described, with URA being associated with a higher risk of genetic syndromes and other extra-urinary malformations and a worse prognosis for long-term renal function compared to unilateral MCDK [55,56].

Prenatal studies focusing on the detection of extra-urinary anomalies in fetuses with URA are scarce. This can be explained by the fact that URA is a rare condition, despite having a true prevalence that is undoubtedly higher than reported due to missed diagnoses in the prenatal period. Clinton and Chasen [57], in one of the few prenatal studies published thus far, studied 102 fetuses with suspected isolated, unilateral renal anomalies and a normal-appearing contralateral kidney. This series included 36 fetuses with URA, 28 with unilateral MCDK, and 38 with renal ectopia. Among those with presumably isolated URA, 4 (11%) had subsequent postnatal findings including VATER syndrome with anal atresia and dysmorphic thumb (*n* = 1), urogenital sinus and colonic atresia (*n* = 1), anal atresia and hypospadias (*n* = 1), and nephrocalcinosis and renal dysplasia (*n* = 1), which highlights the high association with gastrointestinal anomalies in this condition. In another study from Israel, a highly qualified single operator examined 59,382 pregnancies with transvaginal ultrasound between 14 and 16 weeks’ gestation and identified 49 fetuses with URA (prevalence 0.8 per 1000 scans or 1:1212) [58]. Associated anomalies were found in 22 cases (45%), including renal anomalies in 22%, extra-urinary anomalies (excluding isolated single umbilical artery and increased nuchal fold) in 25%, and ambiguous genitalia in 8% of the cases. These investigators concluded that URA is a rare finding in early pregnancy and the rate of associated anomalies is high and worth investigating [58]. The lack of large multicenter prenatal studies precludes accurate information on the true frequency and nature of associated anomalies in the prenatal period, suggesting that additional ultrasound follow-up should be implemented during pregnancy and that postnatal evaluation should be performed for possible associated anomalies. Nephrological and urological consultations are also important to investigate the presence or absence of additional anomalies after birth.

**Figure 6:**
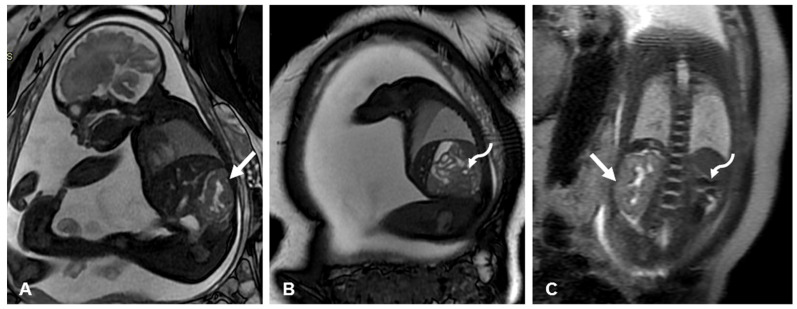
Fetal MRI T2-weighted images in unilateral renal agenesis at 27 weeks’ gestation. (**A**) Right and (**B**) left parasagittal images and (**C**) coronal images of renal fossae. Straight arrow denotes normal kidney. Curved arrow denotes empty renal fossa.

Regarding other prenatal imaging techniques, fetal MRI has been largely used as a complementary technique to evaluate fetuses with complex urinary anomalies, with a high detection yield regarding associated congenital defects [59,60,61]. However, its use in cases of presumably isolated URA seems to be limited although highly accurate (Figure 6). Although MRI may be helpful in assessing the contralateral kidney to rule out ureteral reflux, ureteropelvic junction obstruction, and small atrophic unilateral MCDK or to evaluate an ectopic kidney for its precise location, size, shape, and relationship with the pelvic vessels, this can typically be determined during a detailed ultrasound examination by an experienced operator [3,21].

### 3.2. Prenatal Genetic Evaluation

The prevalence of chromosomal anomalies in fetuses with URA has not been studied in detail as there is no evidence regarding an increased risk of aneuploidy in comparison to normal fetuses or to those with other urogenital anomalies [1]. Indeed, in one of the largest series involving 109 cases of URA diagnosed prenatally and considered to be isolated, no cases of aneuploidy were detected [62]. Therefore, conventional G-banding karyotyping does not seem to be indicated unless other major malformations are found. Sagi-Dain et al. [63] performed chromosomal microarray analysis in 81 fetuses with isolated URA and found 2 (2.5%) with copy number variants and 1 (1.2%) with a variant of unknown significance inherited from a healthy mother; there was no significant difference with a control population. In another study, 120 fetuses with congenital kidney malformations, specifically renal hypoplastic dysplasia including an undetermined number of cases with URA, underwent chromosome microarray analysis. Of the 103 cases of isolated ‘renal hypodysplasia’, as defined in this study, 10 were associated with abnormal results; 3 with URA were found to have abnormal copy number variants, 2 likely pathogenic and 1 of uncertain clinical significance [64]. Similarly, two Chinese groups studied whole-exome sequencing in fetuses with different urological conditions and reported pathogenic variants to be associated with some cases of URA [65,66]. An 11% prevalence of likely pathogenic sequence variants in fetuses with URA was also confirmed by another study performing molecular testing in 9 children with URA [67]. Altogether, these findings suggest that with advances in prenatal genetic testing technology, there seems to be an increasing importance of incorporating these molecular techniques in the prenatal and postnatal evaluation of URA.

## 4. Postnatal Evaluations

### 4.1. Birth Defect Registries

The prevalence of URA has been calculated from information obtained from large population-based birth defect databases (Table 2) [62,68,69]. However, these statistics only include known diagnoses; there are an unknown number of cases missed by neonatal examination due to the asymptomatic nature of URA. Overall, combining these three registries, URA was diagnosed in 674 of the 6,905,061 births, for an incidence of 0.98 per 10,000 births [62,68,69]. From the information available in the latter two registries, 316 (54%) of the cases of URA had associated extra-urinary anomalies.

### 4.2. Early Postnatal Screening with Abdominal/Renal Ultrasound

Overall, URA has been calculated to occur with an incidence of 1/2000 in the general population, whereas the incidence from studies based on prenatal diagnosis is only 1/8000 livebirths [11]. The discordance between the prevalence estimates of URA based on prenatal diagnosis and postnatal diagnosis, as mentioned above, can be attributed to the number of asymptomatic children with URA who do not undergo pediatric abdominal imaging that leads to the diagnosis. Similarly, reports of the frequency with which the contralateral kidney is affected vary by this limitation.

Neonatal screening for congenital anomalies of the kidneys and urinary tract by ultrasound have been conducted by several investigators (Table 3) [70,71,72,73,74]. Most anomalies detected postnatally for the first time were urinary tract dilation or abnormal renal position or parenchyma such as dysplasia, masses, and nephrocalcinosis, but cases of URA were indeed identified. Due to the high prevalence of other congenital anomalies of the kidney and urinary tract, ultrasound screening for urogenital anomalies has been recommended by some, although screening specifically for URA is not recommended.

However, when the prenatal diagnosis of URA is made, an abdominal scan in the neonatal period is highly recommended for several reasons [75,76,77]. First, it will allow for a definitive diagnosis by ruling out other causes of an empty renal fossa. Second, the small possibility of a normally located but severely atrophic and dysplastic kidney can be easily ruled out on a neonatal renal scan. Third, an evaluation of the contralateral kidney for hypertrophy or urinary tract dilation that may be due to vesicoureteral reflux or ureteropelvic junction obstruction can simultaneously be performed, based on which the appropriate follow-up can be planned. In a study of 384 infants from 1 to 3 months of age with URA or unilateral MCDK, Guarino et al. [75] found that the ultrasound measurement of renal length was predictive of the risk of subsequent renal injury. Notably, several studies have determined that evaluation using ultrasound is advantageous over scintigraphy and that the abdominal scan should be the primary imaging modality in these cases [76,77,78].

## 5. Further Pediatric Evaluations

### 5.1. Urinary and Extra-Urinary Anomalies

Both anatomical and functional examinations in children with URA have been conducted by several investigators. Dursun et al. [79] studied 87 children with congenital solitary kidney; 17 cases (19%) were originally detected prenatally, 19 (22%) during an evaluation of other anomalies, and 51 (59%) during an evaluation of urinary tract symptoms. Associated anomalies were detected in 52 children (60%). The most common were urological anomalies, present in 32 (37%) cases. Extra-urinary anomalies were present in 38 children (44%), including cardiac anomalies in 13 (36%), gastrointestinal anomalies in 8 (9%), hematological anomalies in 5 (6%), neurological anomalies in 3 (3%), and other organ anomalies in 9 cases (10%). In a systematic review of 43 studies and 2684 patients, Westland et al. [11] extracted data from 16 studies with a total of 709 cases of URA and found extra-urinary anomalies in 222 patients (31%). Information on the type of anomaly was obtained from 12 studies, and the most frequent were gastrointestinal (16%), cardiac (14%), and musculoskeletal anomalies (13%). Miscellaneous anomalies accounted for the remaining 15%. Kaneyama et al. [80] studied in detail 17 children with URA and found that 11 (65%) had associated anomalies including imperforate anus and VACTERL association and cloaca in 9 (53%). Regarding associated urologic anomalies, 41% had vesicoureteric reflux, 6% had pelviureteric junction stenosis, and 18% had ureterovesical junction stenosis.

Less evident syndromic and non-syndromic congenital anomalies can also be found in cases of URA. Many of them affect reproductive organs and usually present during puberty or early adulthood. These conditions include MURCS (MÜllerian duct aplasia, Renal agenesis, and Cervicothoracic Somite anomalies) syndrome [81], Kallmann syndrome (hypogonadotropic hypogonadism and anosmia) [82], Mayer–Rokitansky–Kuster–Hauser syndrome (underdevelopment of the uterus and upper vagina) [83], Zinner syndrome (URA, seminal vesicle cyst, and ejaculatory duct obstruction) [84,85,86], and Herlyn–Werner–Wunderlich (uterus didelphys, blind hemivagina, and ipsilateral URA), also known as OHVIRA (Obstructed Hemi-Vagina and Ipsilateral Renal Anomaly) syndrome [87].

There is a well-recognized high prevalence rate of Müllerian anomalies in females with URA. A detailed study of the genital tract has revealed uterine anomalies in a number of cases, including findings that can be more significant around puberty [88]. Walawender et al. [89] found a prevalence of 24% of Müllerian anomalies in 221 girls aged 10 years or more with congenital solitary kidney, a five-fold increase compared to unilateral MCDK. In a detailed study by Acien and Acien [90], 276 females between 11 and 65 years of age with genital malformations as the primary diagnosed condition were reviewed. A total of 60 had concomitant URA, and 216 had two kidneys. Among the 60 women with URA, none had a normal uterus in comparison to 14 (7%) in the control group (*p* < 0.05). A bicornis bicollis uterus was found in 27 (45%) versus 13 (6%) (*p* < 0.001), didelphys uterus in 10 (17%) versus 9 (4%) (*p* < 0.001), unicornuate uterus in 10 (17%) versus 27 (12.5%) (NS), bicornis unicollis uterus in 8 (13%) versus 56 (26%) (NS), uterine agenesis or hypoplasia in 4 (7%) versus 13 (6%) (NS), and septated uterus in 1 (2%) versus 25 (12%) (*p* < 0.05), in the study versus the control group, respectively.

Although most investigations have been focused on the well-recognized association of URA and congenital uterine malformations, there are several developmental conditions and co-morbidities that should also be considered in the long-term evaluation of these cases, especially in those involving combined urinary and genital malformations. For example, in infants and children with OHVIRA syndrome, there is a significantly high rate of severe urogenital complications such as hydrocolpos, permanent urinary incontinence, acute urinary retention, and pelvic inflammatory disease [91]. After menarche, hematocolpos, hematometra, hematosalpinx, dysmenorrhea, irregular vaginal bleeding, and endometriosis are also prominent associated conditions. Reproductive issues later in life due to uterine malformations are almost the rule [91].

In males, Zinner syndrome is also a significant condition and a serious cause of infertility due to the resultant hypo/azoospermia in about 45% of affected individuals [84,85,86]. The diagnosis of Zinner syndrome is usually made during the investigation of associated anomalies in cases of URA. Otherwise, the diagnosis is prompted after the study of nonspecific urogenital symptoms triggered by the seminal vesical cyst including pain, prostatism, epididymitis, urinary urgency, dysuria, painful ejaculation, and perineal discomfort. Unfortunately, genital abnormalities including uterine malformations, hypospadias, seminal vesical cyst, and ejaculatory duct obstruction are not likely to be identified during prenatal life despite careful imaging. The diagnosis of Zinner syndrome has also been proven difficult in asymptomatic pediatric patients [85,86].

### 5.2. Renal Function Tests

There are many studies exploring renal function in neonates and children with solitary kidneys shortly after diagnosis. However, the long-term follow-up of children with URA has shown somewhat controversial findings. Schreuder [12] and Schreuder et al. [92] presented and reviewed medical evidence in children with a solitary functional kidney and concluded that the compensatory hyperfiltration phenomenon is associated with an increasing frequency of long-term renal injury evidenced by hypertension, albuminuria, and a reduced glomerular filtration rate in one-third of infants by the age of 10 years and more than 50% by the age of 18 years. A more favorable prognosis was reported by Marzuillo and Polito [93], who found that the prevalence of renal damage was only 4% with a cumulative 94% of children free from renal injury by the age of 17 years. In another study [72], the incidence of albuminuria, hypertension, and a decreased glomerular filtration rate at a median follow-up period of 6.3 years was 3%, 5%, and 1.6%, respectively, consistent with previous results obtained by two other studies including 29 and 22 children, respectively [79,94]. A recent systematic review of the literature confirmed these findings, reporting the risk of proteinuria to be 10% (95% confidence intervals 6.9–14.6), hypertension 7% (95% confidence intervals 5.0–10.9), and/or worsened renal function 8% (95% confidence intervals 5.2–13.4) [95].

Based on these studies and the recent systematic review, we suggest that prenatal counseling includes this increased risk of subsequent renal injury to the remaining kidney and the importance of postnatal follow-up by a pediatric nephrologist. Published recommendations for postnatal evaluation after diagnosis include performing urinalysis to rule out proteinuria, although not routine blood or genetic testing. If compensatory renal growth or a structurally abnormal solitary kidney is detected by ultrasound, plasma creatinine and quantitative proteinuria are indicated [96,97].

## 6. Conclusions

Prenatal examination for the presence and normal appearance and location of both kidneys is an important goal of second- and third-trimester obstetric scans. This evaluation is more easily performed at the time of the second-trimester scan, as the amount of amniotic fluid as well as the dynamic position of the fetus can allow for a direct assessment of both renal fossae with relative ease. Recently, a third-trimester obstetric ultrasound has been advocated, providing an additional opportunity to scan for renal and other congenital abnormalities, particularly those than can evolve with advancing gestation [98].

The first step to diagnosing fetal URA is the examination of both renal fossae for the presence of both kidneys. In cases where an empty renal fossa is detected, the operator should exclude an ectopic kidney and crossed fused ectopia using a multiplanar approach. In addition, a detailed examination of the contralateral kidney is essential to assess for hypertrophic changes or the presence of other urinary anomalies, recognizing that the appearance of the renal parenchyma changes over time and the urinary tract may develop obstruction. The role of color flow imaging in the assessment of renal arteries is also important as this technique can help not only in the prenatal diagnosis of URA but also in the differential diagnosis with ectopic kidney and fused ectopia. Due to the association with extra-urinary abnormalities, prenatal evaluation for anal atresia and genital malformations including hypospadias. If amniocentesis for genetic studies is performed, testing should consist of microarray and possibly whole-exome/genome sequencing. As the diagnosis of URA does not alter obstetric management, parents should be made aware that further investigation should be undertaken during the neonatal period and childhood. These include an evaluation of the kidneys after birth with imaging, a complete assessment of the genital tract, and other investigations including advanced genetic analysis if required. Pediatric nephrology and urology consultations are essential, as well as gynecologic evaluation in females and urologic evaluation in males for potential reproductive issues. These evaluations and timely management may reduce long-term consequences affecting the urinary and reproductive organs.

## Figures and Tables

**Table 1:** Diagnoses in cases of fetal empty renal fossa.

First Author, Year	Cases (*n*)	GA at Diagnosis (Weeks)	URA (*n* (%))	Pelvic Kidney *n* (%)	Horseshoe Kidney *n* (%)	CFRE *n* (%)	Other
Jeanty [28], 1990	6	28-Term	3 (50)	2 (33)		1 (17)	
Yuksel [29], 2004	40	18–37 *	13 (32)	24 (60)	2 (5)	1 (3)	
Chow [30], 2005	93 †	17–39	44 (47)	35 (38)		4 (4)	10 (11) †
Cho [31], 2009	24		12 (50)	6 (25)			6 (25) ‡
Toprak [32], 2021	10	17–24	2 (20)	3 (30)	4 (40)	1 (10)	
Total	173		74 (43)	70 (40)	6 (3)	7 (4)	16 (9)

GA, gestational age; URA, unilateral renal agenesis; CFRE, crossed fused renal ectopia. * Only 18 of the 40 fetuses (45%) were detected before 24 weeks’ gestation. † There were 10 (11%) false positive diagnoses of empty renal fossa, including 7 cases of dysplastic kidneys, 2 cases of normal kidneys, and 1 case in which the kidney was infiltrated by a tumor. ‡ All 6 cases were unilateral ectopic dysplastic kidney.

**Table 2:** Prevalence of unilateral renal agenesis in large population-based birth defect registries.

First Author, Year	Study Period (Years)	Country	Deliveries (*n*)	Cases of URA (*n*)	Prevalence	Isolated/ Non-Isolated
Wilson [68], 1985	1966–1982	Canada	625,132	90	1.4/10,000	NR
Harris [69], 2000	1978–1993 1973–1993 1983–1992	France Sweden USA	1,418,519 2,191,790 2,221,735 =5,832,044 *	=407 *	=0.7/10,000 *	56/56 48/75 58/114 =162/245 *
Laurichesse Delmas [62], 2017	1995–2013	France	447,885	177	4.0/10,000	106/71
Total			6,905,061	674	1.0/10,000	268/316 (46%/54%) †

URA, unilateral renal agenesis. NR, not reported. * Subtotal for Harris et al.’s study. † Excluding Wilson et al.’s study.

**Table 3:** Postnatal screening of unilateral renal agenesis with abdominal/renal ultrasound. Selected studies.

First Author, Year	Population Screened (*n*)	Urinary Anomalies Detected (*n* (%))	URA (*n*)	Characteristics and Age at Screening
Tsuchiya [70], 2003	5700	198 (3.5)	2	Low-risk population; no anomalies detected on prenatal US Routine US at 1 month of age
Gruessner [71], 2012	11,887	335 (2.8)	11	Routine US at 3–10 days of life
Urisarri [72], 2018	32,900	* 128 (0.4)	74	Retrospective observational study in newborn infants. Prenatal diagnosis of URA in 28 (38%) cases and diagnosis in the first week of life in 46 (62%)
Gulyuz [73], 2023	2629	121 (4.6)	9	Infants of 6 weeks to 3 months of age undergoing urinary tract US during routine pediatric care. Anomalies detected antenatally in 75 cases (6 URA) and postnatally in 46 cases (3 URA)
Caiulo [74], 2012	17,783	171 (1.0)	0	Routine US at 2 months of age

URA, unilateral renal agenesis; US, ultrasound. * Only cases of solitary functional kidney were specifically included in the analysis; other urinary anomalies are not reported.

## Data Availability

Data sharing is not applicable to this article as no datasets were generated or analyzed during the current study.
